# Family dissolution and children’s social well-being at school: a historic cohort study

**DOI:** 10.1186/s12887-019-1821-z

**Published:** 2019-12-05

**Authors:** Line Lund Laursen, Kathrine Bang Madsen, Carsten Obel, Lena Hohwü

**Affiliations:** 1 Department of Public Health, Aarhus University, Aarhus, Denmark; 2 Department of Economics, Aarhus University, Aarhus, Denmark

**Keywords:** Family dissolution, Divorce, Parental separation, Social well-being, Ordinary public school, Family structure

## Abstract

**Background:**

Family dissolution has become more common and one third of the child population in most Western countries now experience family dissolution. Studies show that children from dissolved families have lower levels of social well-being than children from intact families, but only few studies have examined the impact on social well-being specifically in the school setting. We investigated the association between family dissolution and children’s social well-being at school, including the possible influence of the child’s age at the time of the family dissolution.

**Methods:**

We defined a historic cohort study of 219,226 children and adolescents aged 9–16 years and combined demographic registry data of family structure with questionnaire data on social well-being based on the Danish National Well-being Questionnaire completed in 2015. The definition of social well-being was constructed on the children’s perception of sense of belonging in the school setting, in the class and the school community, as well as perceptions on safety, loneliness and bullying. We examined low social well-being according to family dissolution and used multiple logistic regression analyses to adjust for parental educational level, ethnicity and siblings and further stratified for gender and age.

**Results:**

A total of 5% of the children had a low social well-being at school. Among the 31% who lived in dissolved families, we found more children with a low level of social well-being at school (adjusted OR 1.41, 95% CI 1.36;1.47) than those in intact families; especially among those who at the time of family dissolution were in the preschool age (1.55, 95% CI 1.47;1.64).

**Conclusion:**

Children from dissolved families had higher odds for low social well-being at school compared with children from intact families, especially those who experienced family dissolution in the preschool age. The school may be an important setting for identifying and providing help and support in children experiencing family dissolution.

## Background

In the past 20 years, family dissolution has become more common in most Western countries and it is estimated that about half of first marriages will be dissolved [1, 2]. A little more than half of all divorces involve children [2]. In 2015, 27% of all children in Denmark under the age of 18 years living at home shared an address with only one parent [3]. In the last decades, several studies have found that children with divorced or separated parents had less favourable outcomes, including academic achievement, psychosocial well-being, self-concept, as well as a higher risk of dropping out of school than children living in intact families [4–7]. These less favourable outcomes in children, which are seen both immediately after the divorce and in a longer perspective, are similar to the outcomes found in interparental conflict [5, 8]. Indeed, conflict levels between parents before, during, and after the parental divorce may explain more about children’s adaptation to parental separation than the actual event of divorce. Interparental conflict may engender attention problems, self-blaming attributions, elevated conflict with peers as well as general emotional and class-room difficulties leading to reduced academic performance in school children [8].

Parents are important resources for the child, providing emotional support, practical assistance and guidance and can serve as role models to teach their children social skills [9]. Thus, the family constitutes a key social setting and, even if parental conflict may not be present, the absence of one parent may be problematic for the child’s socialization [6, 10]. From this perspective, it has been hypothesized that children have a higher level of social well-being if divorce occurs when they are older rather than younger because a considerable part of the socialization process takes place early in the child’s life. Parental dissolution seems to have relatively few consequences for children at college and university age level, presumably because of their maturity and independence from the family [6, 9, 10].

Previous cross-sectional and prospective studies have examined the association between family dissolution and social well-being in children aged 11–18 years. The associations were estimated on sample sizes varying from 978 to 13,953 children and based on various measures reflecting social well-being, including popularity, cooperativeness, peer relations, loneliness, being bullied, perceived social disintegration, and lack of joy in school [6, 7, 11–18]. The majority of these studies found that children from dissolved families have a poorer outcome than children from intact families; a few studies found no association. However, most of the studies were based on self-reported data on family dissolution [6, 7, 11–14, 16, 17] introducing potential bias or they would only include data on families legal dissolution by divorce or separation, leaving out couples who live together but are not married [6, 7, 11, 12, 15, 16, 18]. Furthermore, some studies based social well-being of children on teachers’ or parents’ reporting [6, 7, 14, 18], thus using other informants than the children themselves. Many of the social outcomes in previous studies refers to the school setting, but few of the studies have focused strictly on this particular setting. The school setting is a central part of children’s daily life and may be seen as the single most important social setting outside the home where children spend many hours during a day [19]. In a prospective epidemiological study, poor social well-being has been associated with lower academic performance in school and higher risk of severe mental health problems among a representative sample of 2790 adolescents [20]. The school setting may play a substantial role in early identification of children at risk of poor well-being and in need of support in case of parental separation. Thus, the main objective of this study was to investigate the association between family dissolution and children’s social well-being at school and secondly, to investigate how the association may vary according to the child’s age at the time of the family dissolution. Building on the knowledge in previous studies [4–7], we hypothesized that children from dissolved families had a higher risk of low social well-being at school compared with children from intact families, and that the risk increased the younger the child was at the time of the family dissolution.

## Methods

### Sample

This historic cohort study combined registry data from Statistics Denmark with questionnaire data from the Danish National Well-being Questionnaire in 2015. Since 2015, all public schools in Denmark have completed the National Well-being Questionnaire annually [21]. The National Well-being Questionnaire, specific for children attending 4th–9th grade (ages 9–16 years), consists of 40 questions of which 29 are used by the Ministry of Education to construct four indicators depicting different dimensions of school well-being: Social well-being, academic well-being, support and inspiration in class, and finally classroom silence and order [22]. All children filled in the questionnaire electronically with a personal log-in during school hours alongside with their classmates and with a teacher present [23]. The personal log-in and the unique personal identification number assigned to all citizens in Denmark made it possible to link results of the National Well-being Questionnaire with various national registries. We retrieved historical data about family structure before 2015 in national registries.

**Fig. 1:**
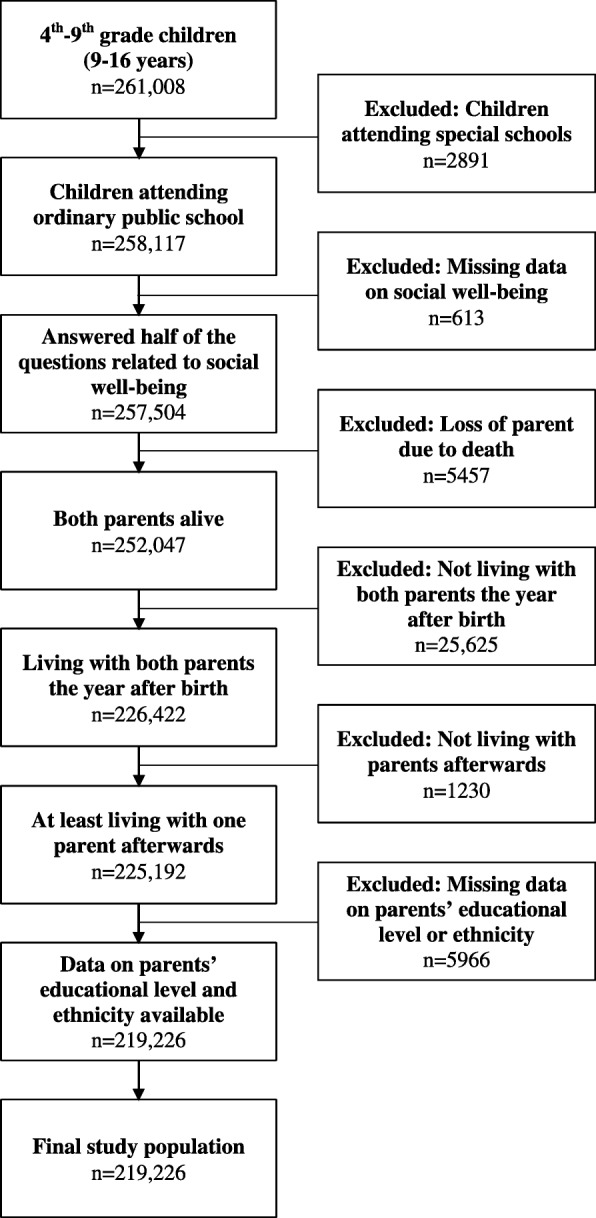
Flowchart of the selection of the study population

In 2015, 314,901 children attended 4th–9th grade in public schools in Denmark [24]. Of these, 261,008 filled in the National Well-being Questionnaire, resulting in a response rate of 83%. Our study excluded children attending special schools (*n* = 2891), children who filled in less than half of the 10 questions by using the option “I don’t want to answer” in the social well-being subscale of the National Well-being Questionnaire (*n* = 613) [25], children who lost a parent due to death (*n* = 5457), children who did not live with both parents the year after birth (*n* = 25,625) and afterwards did not live with at least one parent (*n* = 1230) as well as cases with missing registry data on parental educational level and ethnicity (*n* = 5966). This resulted in a sample of 219,226 children with complete data (Fig. 1).

### Measures

#### Family dissolution

Data on family structure was retrieved from the national register Parent Mark [26]. Family dissolution was defined using an annual mark (estimated on 31st December the previous year) of family structure concerning whether a child was living at the same address as both parents, only one parent or the mother or father who was in a new relationship. If a child in the first year of life was living with both parents on 31st of December and only one parent any following year up until 2015, that constituted family dissolution. Children from dissolved families were compared with children from intact families, where children lived continuously with both parents until 2015.

#### Social well-being

The Ministry of Education has defined a social well-being scale, consisting of 10 questions from the National Well-being Questionnaire (Cronbach’s alpha 0,85) [25]. We used this a priori defined scale that covered a broad spectrum of the children’s perception of social well-being including sense of belonging in the school setting, in the class and the school community, as well as perceptions on safety, loneliness and bullying. The basic psychometric properties of the questionnaire identified high skewness and/or kurtosis in three of the 10 questions: Being bullied, liking the breaks and afraid of being made fun of [27]. Children responded by indicating their level of agreement with each question on a five-point Likert scale, where 1 denoted the worst possible well-being and 5 denoted the best possible. The scores were added and then divided by the number of questions answered, resulting in an average score for each child. The scores were dichotomized into high (≥ 3) and low social well-being at school (< 3).

#### Covariates

Potential confounders were chosen a priori based on previous studies and available registry data on parental educational level, ethnicity, stepparents, changes in family structure and siblings. Parental educational level was reported separately for the mother and father and categorized into three groups based on the number of years of education: Low (≤10), medium (11–14) and high (≥15). Ethnicity was dichotomized into “Danish” consisting of children of ethnic Danish origin and “Immigrant or descendant” consisting of children whose parents did not have Danish citizenship or parents born outside Denmark*.* Siblings were full siblings (No siblings/Siblings).

Four variables were used for stratification. The age of the child at completion of the questionnaire (years) was dichotomized into 9–12 years and 13–16 years approximately equivalent to 4th–6th grade and 7th–9th grade. The age of the child at the time of the family dissolution (years) was categorized into 2–5 years, 6–10 years, 11–16 years, describing pre-school-, early- and late school age. If a child lived with a parent who was in a new relationship this constituted having had a stepparent (Stepparents/No stepparents). Further, the number of changes in the family structure was based on registry data on adults moving in and out of the same address as the child and entered as a categorical variable and coded into “1”, “2” and “> 2”.

### Statistical analysis

Descriptive analyses were conducted to present characteristics of the study population by exposure groups, “Intact family” and “Dissolved family”, and to present characteristics specific for “Dissolved family” regarding the child’s age at the time of family dissolution, stepparents and the number of changes in the family structure. Stratification by age was performed due to statistical interaction. Multiple logistic regression analyses were conducted to estimate unadjusted and adjusted odds ratios (OR) with corresponding 95% confidence intervals (95% CI) of the association between family dissolution and the children’s social well-being at school stratified by the age of the child. A sensitivity analysis using only seven of the 10 questions – leaving out the questions regarding being bullied, liking the breaks and afraid of being made fun of - was conducted following a structure proposed in the previous study assessing the psychometric properties of the questionnaire [27]. Furthermore, analyses dividing “Dissolved families” according to the child’s age at the time of the dissolution were conducted. The analysis was stratified according to registration of stepparents and number of changes in family structure to examine if these would modify the association. In all analyses, derived estimates reflected the risk of a low social well-being at school. Robust standard errors were applied to account for the similarity of siblings in families with full or half siblings on the mother’s side. The analyses were conducted using STATA/MP 14.2 (Stata Corporation, College Stadion, TX, USA).

## Results

### Sample characteristics

Of the study population, 150,433 (69%) children lived in intact families, while 68,793 (31%) lived in dissolved families (Table 1). Among the intact families, more children were 9–12 years when they participated in the survey than in dissolved families. The parents’ educational level was generally higher in intact families than in dissolved families, and more children from intact families had siblings.

**Table 1:** Descriptive statistics of the study population by exposure groups (*n* = 219,226)

	Intact family		Dissolved family	
	*n*	(%)	*n*	(%)
Participants	150,433	(69)	68,793	(31)
Low social well-being at school	6921	(5)	4782	(7)
Gender:				
Boys	76,330	(51)	34,469	(51)
Girls	74,103	(49)	34,324	(49)
Age (years):				
9–12	55,742	(37)	22,179	(32)
13–16	94,691	(63)	46,614	(68)
Mother’s educational level (years):				
≤ 10	15,062	(10)	11,222	(16)
11–14	62,515	(42)	31,666	(46)
≥ 15	73,856	(48)	25,905	(37)
Father’s educational level (years):				
≤ 10	19,779	(13)	14,397	(21)
11–14	71,745	(48)	34,819	(51)
≥ 15	58,909	(39)	19,577	(28)
Ethnicity:				
Danish	138,219	(92)	63,654	(92)
Immigrant or descendant	12,214	(8)	5139	(8)
Siblings:				
No siblings	11,309	(8)	16,285	(24)
Siblings (≥ 1)	139,124	(92)	52,508	(76)

Most children who experienced family dissolution were between 2 and 5 years or 6–10 years at the time of the dissolution (Table 2). Furthermore, the younger the children were at the time of the family dissolution, the more children experienced having stepparents as well as changes in the family structure.

**Table 2:** Descriptive statistics by age of the child at the time of family dissolution (*n* = 68,793)

Age at the time of dissolution (years)	2–5		6–10		11–16	
	*n*	(%)	*n*	(%)	*n*	(%)
*n* (%)	29,024	(42)	27,125	(39)	12,644	(18)
Stepparents:						
No stepparents	12,726	(44)	16,382	(60)	10,730	(85)
Stepparents	16,298	(56)	10,743	(40)	1914	(15)
Number of changes in family structure:						
1	12,217	(42)	15,839	(58)	10,595	(84)
2	14,748	(51)	10,116	(38)	1913	(15)
> 2	2059	(7)	1170	(4)	136	(1)

### Social well-being at school in children from intact families and dissolved families

Children from dissolved families had statistically significantly higher odds for low social well-being at school compared to children from intact families; adjusted OR 1.41 (95% CI 1.36;1.47) (Table 3). The sensitivity analysis removing three of the ten items (being bullied, liking the breaks and afraid of being made fun of) did not alter the OR. Stratification by age revealed that children between 9 and 12 years had significantly but not substantially higher odds of low social well-being at school compared with children between 13 and 16 years; adjusted OR 1.54 (95% CI 1.44,1.64) and 1.36, (95% CI 1.29,1.43), respectively (Table 3).

**Table 3:** Odds ratio for having low social well-being at school (*n* = 219,226)

	Unadjusted		Adjusted^a^	
	OR	95% CI	OR	95% CI
Intact family	1	(reference)	1	(reference)
Dissolved family	1.55	(1.49;1.61)	1.41	(1.36;1.47)
Age 9–12:				
Intact family	1	(reference)	1	(reference)
Dissolved family	1.67	(1.57;1.78)	1.54	(1.44;1.64)
Age 13–16:				
Intact family	1	(reference)	1	(reference)
Dissolved family	1.49	(1.42;1.57)	1.36	(1.29;1.43)

*OR* Odds ratio

*CI* Confidence interval

^a^Adjusted for siblings (no siblings/siblings), ethnicity (Danish/immigrant or descendant), parental educational level (low, medium, high), gender (boy/girl)

We found that the younger the child was when the family dissolved the higher odds for low social well-being at school compared with children from intact families (adjusted OR 1.55, 95% CI 1.47;1.64) (Table 4). When stratified according to stepparents and number of changes in the family structure, we found that children aged 2–5 years at the time of the family dissolution had consistently higher odds of low social well-being compared to older children, except for children aged 11–16 years who had experienced more than two changes in family structure.

**Table 4:** Odds ratio for having low social well-being at school according to child’s age at dissolution (*n* = 219,226)

Age at dissolution	2–5 years		6–10 years		11–16 years	
	OR^a^	95% CI	OR^a^	95% CI	OR^a^	95% CI
Intact family	1	(reference)	1	(reference)	1	(reference)
No stratification	1.55	(1.47;1.64)	1.34	(1.26;1.41)	1.30	(1.20;1.40)
Stratified by stepparent:						
Intact family	1	(reference)	1	(reference)	1	(reference)
No stepparent	1.48	(1.38;1.60)	1.38	(1.29;1.48)	1.28	(1.18;1.39)
Stepparent	1.61	(1.51;1.72)	1.27	(1.17;1.38)	1.38	(1.15;1.66)
Stratified by no. of changes in family structure:						
Intact family	1	(reference)	1	(reference)	1	(reference)
1	1.50	(1.39;1.62)	1.36	(1.27;1.46)	1.29	(1.18;1.40)
2	1.55	(1.45;1.66)	1.27	(1.17;1.39)	1.28	(1.06;1.54)
> 2	1.92	(1.65;2.23)	1.52	(1.21;1.90)	2.47	(1.44;4.24)

*OR* Odds ratio.

*CI* Confidence interval.

^a^Adjusted for siblings (no siblings/siblings), ethnicity (Danish/immigrant or descendant), parental educational level (low, medium, high), gender (boy/girl)

## Discussion

This historic cohort study found that children from dissolved families had increased odds for low social well-being at school compared with children from intact families. Stratification by age revealed that children between 9 and 12 years had higher odds for low social well-being at school than children between 13 and 16 years. Furthermore, the results showed that the younger the child was at the time of the family dissolution, the higher the odds for low social well-being at school.

These findings can be seen as a support of the hypothesis based on the Parental Loss Perspective, which emphasize that the family constitutes a key social setting and the absence of one parent may be problematic for the child’s socialization [6, 9, 10]. Following this, children are expected to have a higher level of social well-being if family dissolution occurs when they are older rather than younger, because a considerable part of the socialization process has already taken place. The importance of the child’s age at family dissolution might also be explained by the younger children from dissolved families may have experienced more changes in family structure, e.g. having stepparents. However, when stratifying for number of changes, the youngest children at the time of the family dissolution had consistently higher odds of low well-being compared to older children.

Parental conflict has been well-documented as the factor explaining most of the negative effects of family dissolution [6, 8, 28]. Unfortunately, we did not have available data on this. If our study aimed at examining family dissolution per se, parental conflict should be adjusted for, and the association in this study would most likely be weaker. Instead, we used family dissolution as an indicator of the process of family dissolution [2]. By adopting a process-oriented perspective on family dissolution, parental conflict should not be adjusted for as it is a substantial part of the process [29].

Our results are in line with previous studies examining family dissolution and various aspects of children’s social well-being [6, 7, 11, 13–15, 17]. However, three studies found no association between family dissolution and children’s social well-being [12, 16, 18] including a Danish and a Norwegian study [12, 16]. The Danish study of 978 adolescents investigated parental divorce alongside with adolescents experiencing change of residence [12]. The study found no significant association between the movers and divorce group and a normative reference group in relation to perception of peer-related loneliness. The Norwegian study of 4127 students aged 11–15 found no significant association between children living with divorced single mothers and social disintegration when compared with children living in intact families [16]. The study did, however, find an association between girls living with divorced single mothers and being bullied. The definition of exposure groups in both studies differed from our study. Furthermore, the information of family dissolution only included family dissolution by legal divorce and was based on self-reports and thus introducing potential bias. A possible explanation for the null-findings in the aforementioned studies has its origin in The Stress Relief Hypothesis introduced by Wheaton (1990) and contends that a stressful life event may actually have beneficial effects on children when divorce is an escape from a harmful, high-conflict environment [30]. Furthermore, according to the findings of Wallerstein and Kelly, the school was a sanctuary for some children of divorced parents [31]. Our findings did not support these possible explanations.

### Strengths and limitations

The major strengths of our study were that our analyses were based on a population sample extracted from national registries with full set of variables, enhancing statistical strength and eliminating recall bias and exposure misclassification. Furthermore, questionnaire data on social well-being were reported by the children themselves and not by teachers or parents as in previous studies [6, 7, 14, 18]. Teachers and parents might not be suitable to report children’s perspective since they only have a partial picture of the child. The teachers are unable to evaluate the child’s well-being outside the class setting and the parents are unable to evaluate the child’s well-being in a school setting. Furthermore, teachers may only meet the children during lessons [32]. Support for using children as informants was found in a study concluding that teachers judge a child of divorce less on the basis of his/her observed behaviour and more on the basis of preconceived stereotype expectations to a child of divorced parents [33]. Also, a meta-analysis found that effect sizes based on the reports of parents and teachers tended to be weaker than effect sizes based on reports from children. This suggests that parents and teachers either underestimate the children’s problems [6] or that children exaggerate their problems. Only children attending 4th–9th grade in ordinary public schools were included, as the questionnaire is considered more valid from the 4th grade [34]. In addition, excluding children who lost a parent due to death and children not living with any parent e.g. children placed in care, maintained the focus of the study on the influence of family dissolution.

The study has some limitations. The first is related to family dissolution being constructed using an annual registration of family structure estimated at the 31st of December the previous year. The only knowledge of family structure in the year of the child’s birth was the one applicable on 31st December where the child had to live with both parents to be included in the study. This entailed that a child who lived with both parents from birth but experienced family dissolution before the 31st of December in the first year of life would not be included. Unfortunately, information of how many children this applies to was not available, but approximately 4 % of parents in Denmark separate when the children are 1–2 years old [35]. Furthermore, it was only possible to track one change in family structure per year in the registries, reducing the validity of this particular variable. The reduced accuracy of family dissolution also affected the accuracy of the child’s exact age at the time of the family dissolution, leaving room for small variations. Using family dissolution as an indicator of parental break-up as opposed to divorce may, however, be viewed as a strength, because people may live together without being married. However, circumstances such as living apart due to work conditions while being in a continuous relationship should also be considered.

A second limitation relates to social well-being at school measured on a scale that has not yet been validated. Thus, ability of the scale to detect true positive and true negative cases with the particular cut-off value in the study is unknown. As a result, there is a risk of outcome misclassification. However, since the possible misclassification most likely did not depend on exposure it would be non-differential implying bias towards the null hypotheses. A study assessing the psychometric properties of the questionnaire proposed a different four-factor structure [27]. However, our sensitivity analysis did not change the results, supporting the internal validity of the scale used to measure social well-being in this study. The scale aimed at measuring social well-being in a school context, thus the construct validity of the scale depended on whether or not the children’s answers related to their social well-being at school and not their social well-being in general. We were unable to test this. The framing of the questions by including “school” could suggest that the validity was not compromised. Only the question about loneliness did not guide the child to focus on the school setting, thus leaving room for answers about general loneliness. The validity might be compromised because of the questionnaire being filled in while being among classmates. This phenomenon has been seen in interviews with children who would avoid answering questions, that they did not want their classmates to know the answers of [36]. Assuming this was due to low social well-being at school, this could indicate two problems. If one way of avoiding answering was to underreport their actual problem, it would most likely be independent of their exposure status causing non-differential outcome misclassification. If another way of avoiding answering was to use the option “I don’t want to answer”, this would increase the likelihood of being excluded. Assuming excluded children had low social well-being at school, this would entail selection bias if exclusion of children also depended on exposure. A significant difference in the distribution of exposure group was found among included and excluded children, where family dissolution was more common among excluded children (not shown). Thus, the study was most likely subject to selection bias causing underestimation of the association. Selection bias could also be apparent in children not filling in the questionnaire. Unfortunately, this could not be investigated as no data were available.

Even though the use of registry data was a major strength of this study, it should be noted that the data were collected for administrative purposes or solely in order to produce public statistics and not for research purposes [37]. Some registry data on parents’ education and ethnicity were missing, but there was no reason to believe that the missing data would result in selection bias as it did not depend on exposure status.

As we used data from the first National Well-being Questionnaire it was not possible to adjust for prior social well-being at school, i.e. if the children from dissolved families had low social well-being before the family dissolution. However, if data on prior social well-being at school was available, the question of whether it should be adjusted for emerges. A study found evidence of children being affected by the disruption process at least 2–4 years prior to the actual family dissolution, possibly as a result of parental conflicts [6, 28, 29].

## Conclusion

In conclusion, this study found that children experiencing family dissolution had a significantly higher risk of low social well-being at school compared with children from intact families. Furthermore, the younger the child was at the time of the family dissolution, the higher the risk of low social well-being at school.

The school may be an important setting where children at risk of poor well-being as a result of parental separation can be identified and receive help and support. Here, the health visitors in collaboration with a pedagogical and psychological consulting team could be central; example by offering group sessions to children who experience family dissolution. Future studies should address the importance of the child’s age at the time of the family dissolution as well as include possible predictors of the increased risk among the youngest age group to improve identification and support of these children.

## Data Availability

The datasets in the current study are not publicly available in accordance with Danish legislation.

## Abbreviations

CI: Confidence interval
OR: Odds ratio
